# Significantly Enhanced Acidic Oxygen Evolution Reaction Performance of RuO_2_ Nanoparticles by Introducing Oxygen Vacancy with Polytetrafluoroethylene

**DOI:** 10.3390/polym17010059

**Published:** 2024-12-29

**Authors:** Jinyang Zhang, Xinru Wang, Xinyue Zhao, Honglei Chen, Peng Jia

**Affiliations:** 1 Key Laboratory of Pulp and Paper Science & Technology of Ministry of Education/Shandong Province, State Key Laboratory of Biobased Material and Green Papermaking, Faculty of Light Industry, Qilu University of Technology (Shandong Academy of Sciences), Jinan 250353, China; zhangjy@nankai.edu.cn (J.Z.); 202291040040@stu.qlu.edu.cn (X.W.); 202391053009@stu.qlu.edu.cn (X.Z.); 501220@qlu.edu.cn (H.C.); 2 Key Laboratory of Advanced Energy Materials Chemistry (Ministry of Education), Renewable Energy Conversion and Storage Center (RECAST), College of Chemistry, Nankai University, Tianjin 300071, China

**Keywords:** oxygen evolution reaction (OER), water splitting, RuO_2_, polytetrafluoroethylene

## Abstract

The supported RuO_2_ catalysts are known for their synergistic and interfacial effects, which significantly enhance both catalytic activity and stability. However, polymer-supported RuO_2_ catalysts have received limited attention due to challenges associated with poor conductivity. In this study, we successfully synthesized the RuO_2_-polytetrafluoroethylene (PTFE) catalyst via a facile annealing process. The optimized nucleation and growth strategies enable the formation of RuO_2_ particles (~13.4 nm) encapsulating PTFE, establishing a conductive network that effectively addresses the conductivity issue. Additionally, PTFE induces the generation of oxygen vacancies and the formation of stable RuO_2_/PTFE interfaces, which further enhance the acidic OER activity and the stability of RuO_2_. As a result, the RuO_2_-PTFE catalyst exhibits a low overpotential of 219 mV at 10 mA cm⁻^2^ in the three-electrode system, and the voltage of the RuO_2_-PTFE||commercial Pt/C system can keep 1.50 V for 800 h at 10 mA cm^−2^. This work underscores the versatility of PTFE as a substrate for fine-tuning the catalyst morphology, the crystal defect, and the stable interface outerwear. This work not only broadens the application scope of PTFE in catalyst synthesis but also provides a novel approach to the design of high-performance metallic oxide catalysts with tailored oxygen vacancy concentration and stable polymer outerwear.

## 1. Introduction

Sustained reliance on non-renewable energy sources often leads to energy shortages and environmental pollution. In light of the abundant water resources on Earth, the use of renewable energy to decompose water into hydrogen can achieve zero carbon emissions. Consequently, it is expected to play a pivotal role in future energy strategies to produce hydrogen by using renewable energy sources. Commercial alkaline water electrolysis, a widely used method, suffers from low energy efficiency, low hydrogen purity and significant operational risks [1]. As a result, the proton exchange membrane water electrolyzer (PEMWE) has garnered considerable attention due to its high energy efficiency, exceptional hydrogen purity, and significant potential for industrial application [2]. However, the advancement of PEMWE faces challenges related to anode catalysts. Presently, only Ir- and Ru-based catalysts demonstrate both high intrinsic activity and oxidation resistance [3]. The scarcity of Ir renders it significantly more expensive than Ru. While Ru offers a more affordable alternative, it fails to meet the stringent corrosion resistance requirements which are necessary for the oxygen evolution reaction (OER). This underscores the urgent need for the development of acid-stable OER catalysts. The current strategies for enhancing Ru-based catalysts include doping and heterojunction engineering, such as Cr-doped RuO_2_ [4], MRuO_x_ (M = Sc, Ti, V, Cr, Mn, Fe, Co, Ni, Cu, Zn) [5], RuO_2_/graphene [6], Pb-RuO_2_ [7], and RuO_2_/CoO_x_ [8]. The incorporation of additional metal elements has been proven as an effective strategy for improving the activity and stability of Ru-based catalysts. However, the potential of combining Ru-based catalysts with anti-corrosion polymers remains underexplored. The primary limitation lies in the poor electrical conductivity of polymers despite their excellent acidic corrosion resistance.

Polytetrafluoroethylene (PTFE), a corrosion-resistant polymer widely employed as a binder in acidic and alkaline electrodes, is hindered in the electrocatalytic field by its negligible electrical conductivity. To address this issue, we developed a novel and straightforward approach to fabricate a RuO_2_-PTFE catalyst. In this approach, we introduce PTFE to induce the formation of a conductive network, oxygen vacancies, and stable RuO_2_/PTFE interfaces. Benefiting from the above merits, the RuO_2_-PTFE catalyst exhibits high acidic OER activity and long-term stability. This innovative design holds promise for advancing the development of acid-stable and efficient catalysts for PEMWE applications.

## 2. Materials and Methods

The RuO_2_-PTFE was synthesized in a one-step process: RuCl_3_ (0.10 mmol) was dissolved in 1 mL of 5% PTFE aqueous solution at the ambient temperature, and then the solution was placed into a muffle furnace for 6 h at 350 °C with a temperature ramping rate of 5 °C min^−1^. After annealing, the black powder is named RuO_2_-PTFE. For comparison, the RuO_2_-F was synthesized at the target annealing temperature of 450 °C, according to the above procedures.

The morphologies and microstructures of RuO_2_-PTFE and RuO_2_-F were characterized by a field emission scanning electron microscopy (JEOL JSM-7500F/JSM-7900F, Tokyo, Japan) and a high-resolution transmission electron microscopy (HRTEM, Talos F200X G2, AEMC, Waltham, MA, USA). The crystal structures were determined by a powder X-ray diffractometer (XRD, Rigaku MiniFlexII, *λ* = 0.154 nm, Tokyo, Japan). The surface chemical compositions and elemental valences were analyzed by an X-ray photoelectron spectroscopy (XPS, Thermo Fisher, ESCALAB250XI, Waltham, MA, USA). The online differential electrochemical mass spectrometry (DEMS) spectra were recorded by a mass spectrometer (Linglu QAS 100, Shanghai, China). The presence of O vacancies was confirmed using electron paramagnetic resonance (EPR, Bruker A300, Rheinstetten, Baden-Württemberg, Germany) spin-trapping measurement. The N_2_ ad-/desorption isotherms obtained from a surface area and porosity analyzer (TriStar II 3020, Atlanta, GA, USA) were used to calculate the specific surface area (SSA) and the pore size distribution.

Before performing the three-electrode test, a catalyst ink with a concentration of 10 mg mL^−1^ was prepared. This involved mixing 5 mg of catalyst with 450 μL of ethanol and 50 μL of Nafion solution (5% concentration, Alfa Aesar), followed by 30 min of continuous ultrasonic treatment to ensure uniform dispersion. The resulting ink was then drop cast onto a glassy carbon (GC) electrode with a diameter of 5 mm and allowed to dry naturally. The loading amount of catalyst on the electrode was ~0.2 mg cm^−2^, making it ready for electrochemical testing. A Hg/Hg_2_SO_4_ electrode and a carbon rod were used as the reference and counter electrodes, respectively. The electrolyte solution was 0.5 M of H_2_SO_4_. The linear sweep voltammetry (LSV) was conducted at a scanning rate of 5 mV s⁻¹, covering a potential range from 1.1 to 1.6 V versus the reversible hydrogen electrode (RHE). To assess durability, the accelerated degradation test (ADT) involving 10,000 voltammetry cycles was conducted. For the overall water-splitting experiments, the anode and cathode electrodes were loaded with 1 mg cm^−2^ of RuO_2_-PTFE and Pt/C on carbon cloth, respectively. The potential was calibrated to the reversible hydrogen electrode (RHE) using the following equation: E_RHE_ (V) = E(Hg/HgSO_2_) + E_0_ + 0.0597 × pH.

## 3. Results

**Figure 1:**
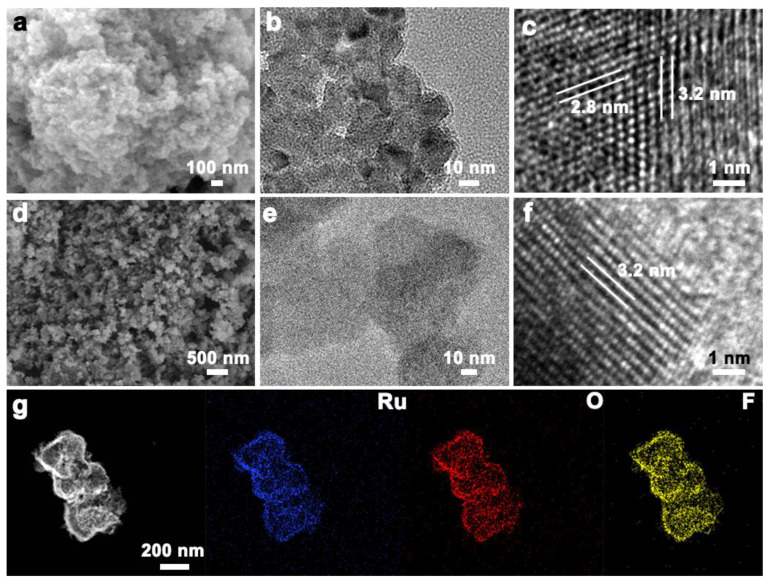
(**a**–**c**) SEM, TEM, and HRTEM images of RuO_2_-PTFE; (**d**–**f**) SEM, TEM, and HRTEM images of RuO_2_-F; (**g**) STEM image and corresponding Ru, O, and F mapping images of RuO_2_-PTFE.

In the electrochemical field, the PTFE is commonly used as a binder but is generally ineffective as a catalyst support due to its high viscosity and low conductivity. However, its excellent corrosion resistance makes it a promising material for applications in acidic oxygen evolution reaction (OER) processes, potentially addressing catalyst stability challenges. To leverage the corrosion resistance of PTFE while keeping the high conductivity of RuO_2_, a novel RuO_2_-PTFE catalyst was synthesized through a simple and controllable annealing process. When annealed at 350 °C, PTFE nanoparticles remain intact and act as nucleation centers for RuO_2_, becoming encapsulated by RuO_2_ nanoparticles. This process leads to the formation of the RuO_2_-PTFE catalyst. In contrast, at 450 °C, the growth of RuO_2_ resulted in the formation of a separate RuO_2_ phase, termed RuO_2_-F. In SEM images (Figure 1a,d) at 350 °C, the formed RuO_2_ nanoscale particles completely enclose the PTFE, whereas at 450 °C, the RuO_2_ particles are bigger and more separated. TEM images (Figure 1b,e and Figure S1) further corroborate these observations, showing distinct particle morphologies. RuO_2_ nanoparticles in RuO_2_-PTFE are interconnected, establishing a conductive network, whereas RuO_2_ nanoparticles in RuO_2_-F are dispersed. The SSAs of RuO_2_-PTFE and RuO_2_-F are ~61.9 and ~33.9 m^2^ g^−1^ (Figure S2a). The pore diameter sizes of RuO_2_-PTFE and RuO_2_-F are ~17.8 and ~3.8 nm (Figure S2b), suggesting the higher exposed area and more active sites of RuO_2_-PTFE, which contribute to improving the OER activity. In addition, the average particle sizes of RuO_2_-PTFE and RuO_2_-F are ~13.4 nm and ~43.6 nm (Figure S3), which is calculated by the statistical average of RuO_2_ nanoparticles in TEM images (Figure S1). Meanwhile, the lattice spacings are ~0.32 nm and ~0.28 nm (Figure 1c,f), which are the (110) and (101) crystal planes of the RuO_2_ phase (PDF# 43-1027). The elemental mapping analysis (Figure 1g) shows a uniform composition distribution of Ru, O, and F across the RuO_2_-PTFE catalyst, indicating the broad RuO_2_/PTFE interface interaction. This homogeneous dispersion of the RuO_2_/PTFE interface enhances the stability of the RuO_2_-PTFE catalyst under acidic OER conditions, making it a promising candidate for durable catalytic applications.

**Figure 2:**
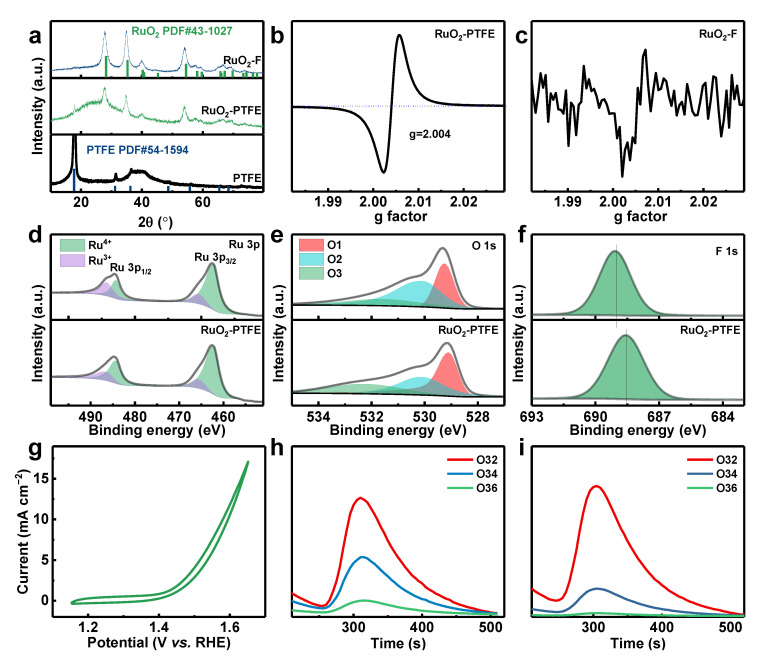
(**a**) XRD patterns of PTFE, RuO_2_-PTFE, and RuO_2_-F; EPR spectra of (**b**) RuO_2_-PTFE and (**c**) RuO_2_-F; high-resolution XPS spectra of (**d**) Ru 3p, (**e**) O 2p, and (**f**) F 1s for RuO_2_-PTFE and RuO_2_-F; (**g**) CV curve of RuO_2_-PTFE for DEMS test; DEMS spectra of (**h**) RuO_2_-PTFE and (**i**) RuO_2_-F.

To further investigate microstructure features, the XRD patterns of RuO_2_-PTFE and RuO_2_-F are displayed in Figure 2a. The XRD pattern has a prominent peak at 18°, which belongs to the PTFE (PDF#54-1594). This peak can also be found in XRD patterns of RuO_2_-PTFE. However, it is nearly absent in RuO_2_-F. This indicates that the supported PTFE is indeed present in the RuO_2_-PTFE catalyst, which is helpful for forming an electron network by connecting RuO_2_ nanoparticles to improve the OER activity. The oxygen vacancies of RuO_2_-PTFE and RuO_2_-F are investigated, and the EPR spectra are depicted in Figure 2b,c. Obviously, the oxygen vacancies appear in the RuO_2_-PTFE catalyst and disappear in the RuO_2_-F catalyst, suggesting that the supported PTFE induces the formation of oxygen vacancies in the RuO_2_ phase. To study the potential of catalysts in acid OER, the chemical composition and group functionality were measured by XPS, and the high-resolution spectra of Ru 3p, O 1s, and F 1s are shown in Figure 2d–f. The Ru 3p can be divided into four peaks corresponding to Ru^3+^ (465.8 and 486.5 eV) and Ru^4+^ (462.5 and 484.4 eV) [9]. The ratio of Ru^3+^/Ru^4+^ in RuO_2_-PTFE is 42:100 higher than that in RuO_2_-F (35:100), which is opposite to the result in Reference [10]. As reported in reported references, oxygen vacancies can provide low-valence Ru ions to improve stability in the acid OER process. This abnormal result mainly depends on the formation of the RuO_2_/PTFE interface with strong interaction, which will be elaborated in detail in the F 1s XPS spectrum. Regarding the O 1s, the peaks of O1 (529.3 eV), O2 (530.2 eV), and O3 (531.7 eV) are fitted to represent the lattice O, OH_ads_, and surface water, respectively [5,11]. The content of O1 in RuO_2_-PTFE is 32.4% lower than that in RuO_2_-F (35.6%), further suggesting the existence of oxygen vacancies in RuO_2_-PTFE. Interestingly, the F 1s spectrum has an apparent red shift in RuO_2_-PTFE (688.6 eV) compared with RuO_2_-F (689.1 eV), indicating the existence of PTFE [12,13] and the RuO_2_/PTFE interface with strong interaction. The strong interaction leads to the higher valence state of Ru ions. Thus, the strong interaction has a more significant impact on the valence state of Ru ions than oxygen vacancies. The content of F is also calculated by XPS spectra, and the values are 6.1 at% and 2.2 at% for RuO_2_-PTFE and RuO_2_-F, respectively. This result indicates that the broader RuO_2_/PTFE interface exists in the RuO_2_-PTFE catalyst, which is conducive to improving the OER stability of the catalyst.

Furthermore, the influence of oxygen vacancies has a significant effect on the acid OER mechanism. The differential electrochemical mass spectrometry (DEMS) was employed to investigate this, as shown in Figure 2g–i. The cyclic voltammetry (CV) curve of RuO_2_-PTFE was used to test DEMS (Figure 2g). After 200 s, the signal stabilizes, and the CV and DEMS are run simultaneously. The DEMS spectra for RuO_2_-PTFE and RuO_2_-F are different (Figure 2h,i). Compared to RuO_2_-F, RuO_2_-PTFE has a lower O^16^O^18^ (O34)/O^16^O^16^ (O32) content (20.6%) than that of RuO_2_-F (50.8%), indicating that the lower content of lattice oxygen for RuO_2_-PTFE participates in the OER process. Thus, the acid OER mechanism of RuO_2_-PTFE is the adsorbate evolution mechanism (AEM) and is displayed in Figure S4. In detail, the Ru active sites of RuO_2_-PTFE first serve as adsorbed sites of water molecules (H_2_O). Secondly, the absorbed H_2_O molecules begin to decompose and oxidize on the Ru active sites in the following sequence: H_2_O_ads_ → * OH → * O → * OOH → O_2_. In a word, the acidic OER activity and stability of RuO_2_-PTFE catalyst are enhanced by the presence of PTFE.

**Figure 3:**
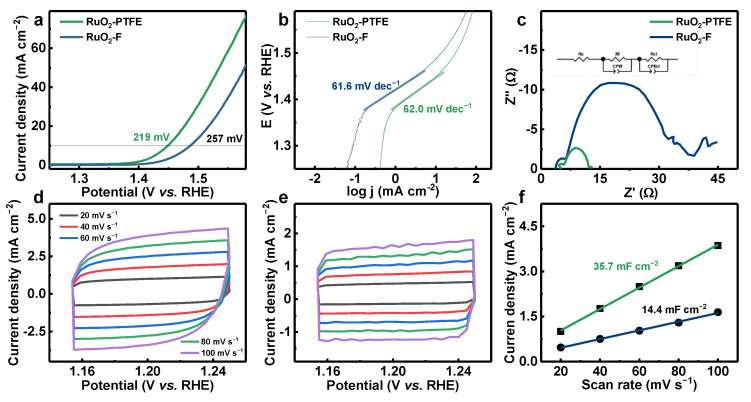
(**a**) LSV curves; (**b**) Tafel plots; (**c**) Nyquist plots; CV curves of (**d**) RuO_2_-PTFE and (**e**) RuO_2_-F; (**f**) C_dl_ calculated by (**d**,**e**) in the three-electrode system.

The OER performances of RuO_2_-PTFE and RuO_2_-F were evaluated by using a three-electrode system. It can be found from the linear sweep voltammetry (LSV) curves (Figure 3a) that RuO_2_-PTFE exhibits an overpotential of ~219 mV, significantly lower than ~257 mV (RuO_2_-F) and RuO_2_ (~294 mV) [10], indicating superior OER activity. As depicted in the Tafel plots (Figure 3b), the Tafel slopes are ~62.0 mV dec^−1^ for RuO_2_-PTFE and ~61.6 mV dec^−1^ for RuO_2_-F, suggesting the comparable reaction kinetics for both catalysts. As shown in the Nyquist plots (Figure 3c), two semicircles displayed in both catalysts and the inserted equivalent circuit reveal two distinct charge transfer processes. The first semicircle at high frequencies corresponds to catalyst oxidation, while the second semicircle represents the acidic OER [14]. Notably, RuO_2_-PTFE exhibits a lower charge transfer resistance (~6.3 Ω) than RuO_2_-F (~26.6 Ω), which enhances the electron transfer rate of RuO_2_-PTFE due to its unique crystal defect and RuO_2_-PTFE interfaces. To estimate the number of intrinsic active sites, the electrochemical double-layer capacitance (C_dl_) was calculated from cyclic voltammetry (CV) curves at different scan rates in the non-Faradaic potential range of 100 mV (Figure 3d,e). As shown in Figure 3f, RuO_2_-PTFE achieves a C_dl_ of ~35.7 mF cm^−2^, 1.5 times more than RuO_2_-F (~14.4 mF cm^−2^), indicating that the electrochemically active surface areas of RuO_2_-PTFE and RuO_2_-F are ~892.5 and ~360.0 cm^2^ per a geometric area of 1.0 cm^2^, respectively. This demonstrates that RuO_2_-PTFE possesses a significantly higher density of active sites. As for the stability, the accelerated degradation test (ADT) was conducted by the 10,000 CV. Before and after the ADT, the LSV curves of RuO_2_-PTFE and RuO_2_-F were recorded and exhibited in Figure S5. After 10,000 CV, the overpotential of RuO_2_-PTFE has a nearly negligible change (Δη ≈ 0 mV), which is far lower than that of RuO_2_-F (Δη = 31 mV). Moreover, the TEM image of RuO_2_-PTFE still exhibits apparent lattice spacing for RuO_2_ (110) after the ADT (Figure S6), indicating that RuO_2_-PTFE can keep stability during the ADT. In addition, the remarkable OER activity of RuO_2_-PTFE is higher than that of other reported references [4,15,16,17,18,19,20] (Table S1), which can be attributed to its abundant vacancies and the high concentration of active sites. In short, RuO_2_-PTFE is a promising candidate for the highly efficient and long-term stable catalyst for acidic OER application.

**Figure 4:**
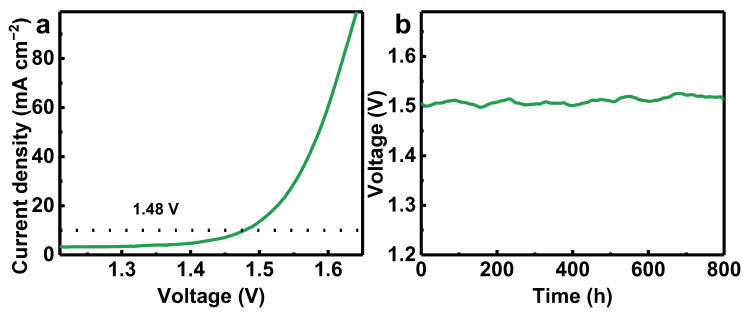
(**a**) Two-electrode performance of commercial RuO_2_-PTFE||commercial Pt/C catalysts; (**b**) the curves of voltage versus time for the galvanostatic stability test.

The application potential of RuO_2_-PTFE was also studied on a two-electrode system: RuO_2_-PTFE as an anode and the commercial Pt/C as a cathode. As shown in the LSV curve (Figure 4a), the two-electrode system exhibits a voltage of 1.48 V at 10 mA cm^−2^. Additionally, the voltage of the two-electrode system can keep 1.50 V for 800 h at a galvanostatic condition of 10 mA cm^−2^ (Figure 4b). Based on the above results, the two-electrode system exhibits high activity and excellent stability. Therefore, RuO_2_-PTFE has good potential for acidic OER commercial applications.

## 4. Conclusions

In this study, we prove the potential of PTFE as a versatile substrate for tailoring catalyst morphology, crystal defects, and stable interfaces. Concretely, the incorporation of PTFE induces the formation of oxygen vacancies, stable RuO_2_/PTFE interfaces and a conductive network that overcomes the traditional conductivity challenges, leading to enhanced acidic OER activity and the stability of the RuO_2_-PTFE catalyst. The resulting RuO_2_-PTFE catalyst exhibits impressive performance, with a low overpotential of 219 mV at 10 mA cm⁻^2^ in a three-electrode system and a sustained voltage of 1.50 V for 800 h at 10 mA cm^−2^ in a RuO_2_-PTFE||commercial Pt/C system. This work not only highlights the RuO_2_-PTFE catalyst as a promising candidate for the acidic OER application but also provides a novel strategy for designing high-performance metallic oxide catalysts with controlled oxygen vacancy concentrations and stable polymer coatings.

## Data Availability

The data presented in this work are available on request from the corresponding author.

## Supplementary Materials

The following supporting information can be downloaded at: https://www.mdpi.com/article/10.3390/polym17010059/s1, Figure S1: TEM images of (a) RuO_2_-PTFE and (b) RuO_2_-F; Figure S2: (a) N_2_ ad-/desorption isotherms and (b) pore size distribution plots of RuO_2_-PTFE and RuO_2_-F; Figure S3: The particle size distributions of (a) RuO_2_-PTFE and (b) RuO_2_-F, which are calculated by the TEM image in Figure S1; Figure S4: The schematic diagram of the OER mechanism for RuO_2_-PTFE; Figure S5: The LSV curves of (a) RuO_2_-PTFE and (b) RuO_2_-F before and after the 10,000 CV accelerated degradation test (ADT); Figure S6: TEM image of RuO_2_-PTFE after ADT; Table S1: Comparison in OER activity with the recently reported Ru-based electrocatalysts in acidic conditions. (References [1,4,5,6,7,15,16,17,18,19,20] are also cited in Supplementary Materials).
