# Supplementary material for: Significantly Enhanced Acidic Oxygen Evolution Reaction Performance of RuO2 Nanoparticles by Introducing Oxygen Vacancy with Polytetrafluoroethylene
Source: Polymers (Basel). 2024 Dec 29;17(1):59. doi: 10.3390/polym17010059 (PMC11723143; doi:10.3390/polym17010059)
Supplement: Supplementary file 1 [file polymers-17-00059-s001.zip › polymers-3378037-supplementary.pdf]

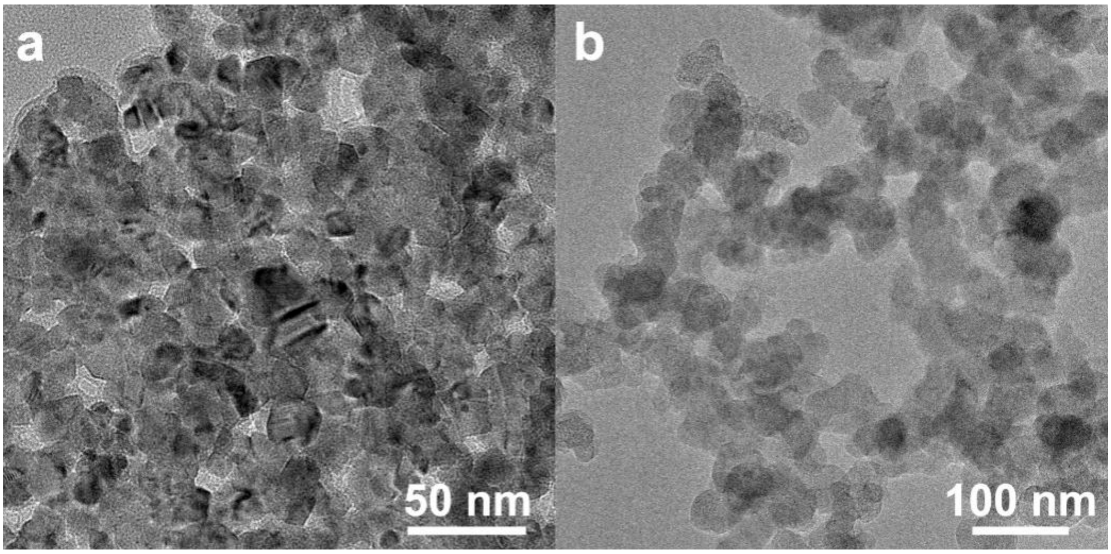

Figure S1. TEM images of (a) RuO_2-PTFE and (b) RuO_2-F.

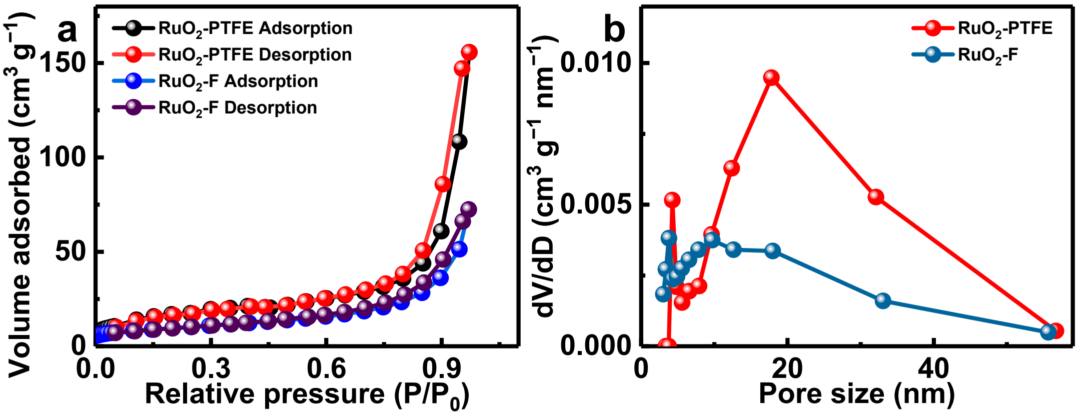

Figure S2. (a) N_2 ad-/desorption isotherms and (b) pore size distribution plots of RuO_2-PTFE and RuO_2-F.

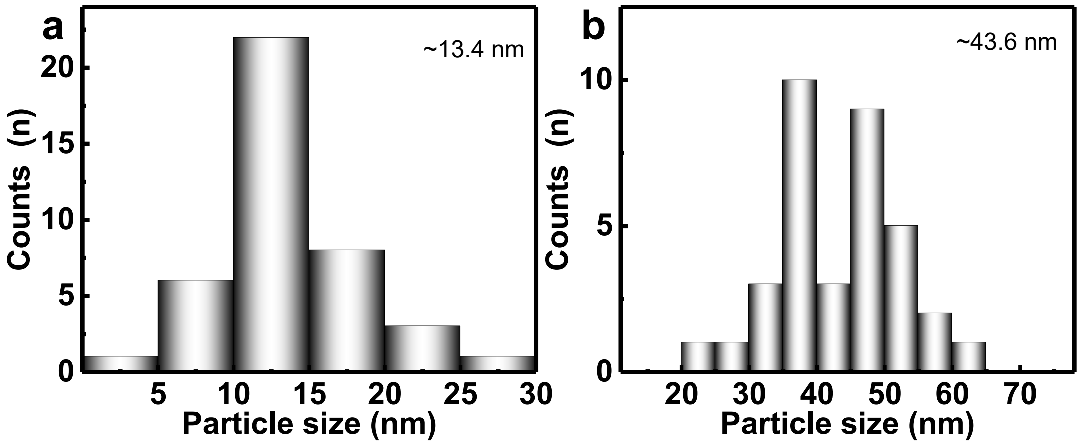

Figure S3. The particle size distributions of (a) RuO_2-PTFE and (b) RuO_2-F are calculated by the TEM image in Figure S1.

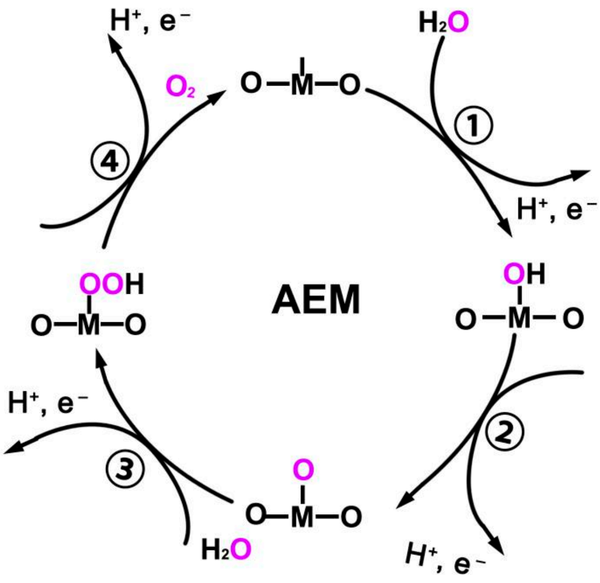

Figure S4. The schematic diagram of the OER mechanism for RuO_2-PTFE.

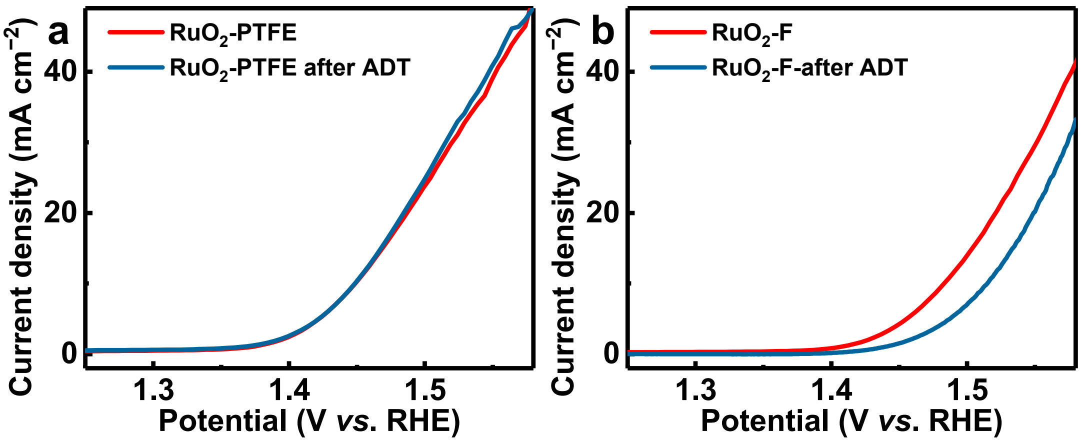

Figure S5. The LSV curves of (a) RuO_2-PTFE and (b) RuO_2-F before and after the 10000 CV accelerated degradation test (ADT).

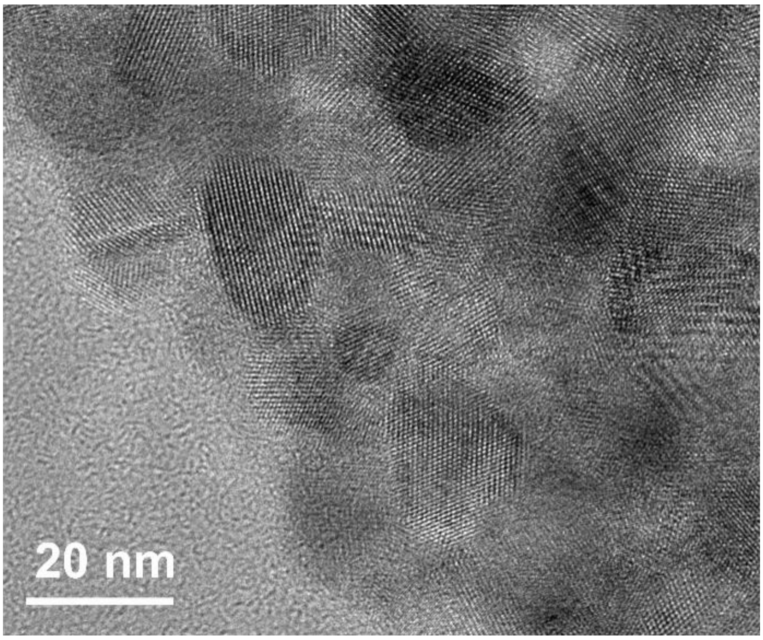

Figure S6. TEM image of RuO_2-PTFE after ADT.

Table S1: Comparison in OER activity with the recently reported Ru-based electrocatalysts in acidic conditions.

Sample	Loading (mg cm^{-2})	Overpotential (mV) @10 mA cm^{-2}	Tafel slope (mV dec^{-1})	References
**RuO_2-PTFE**	**0.20**	**219**	**62.0**	**This work**
**RuO_2-F**	**0.20**	**257**	**61.2**	**This work**
RuO_2 NSs	0.13	199	38.2	[S1]
Hm-RuO_2	0.50	245	85.7	[S2]
(Com)-RuO_2	0.50	280	97.9	[S2]
$Ru_{1-x}O_2$	0.25	212	70.4	[S3]
RuO_{2-x}	0.25	243	81.9	[S3]
RuO_2	0.25	276	81.5	[S3]
Commercial RuO_2	0.25	328	112.3	[S3]
RuO_2	0.40	363	~	[S4]
Com. RuO_2	1.00	285	82.4	[S5]
Com. RuO_2	0.28	300	68.3	[S6]
RuO_2	0.20	223	66.8	[S7]
RuO_x	0.25	300	81.0	[S8]
RuO_2	~	242	72.7	[S9]
RuO_2	0.50	260	59.9	[S10]
p-RuO_2	0.34	270	114.0	[S11]

References

[S1] Zhao, Z.L.; Wang, Q.; Huang, X.; Feng, Q.; Gu, S.; Zhang, Z.; Xu, H.; Zeng, L.; Gu, M.; Li, H. Boosting the oxygen evolution reaction using defect-rich ultra-thin ruthenium oxide nanosheets in acidic media. *Energy Environ. Sci.* **2020**, *13*, 5143-5151.
DOI: 10.1039/d0ee01960g

[S2] Zhou, C.H.; Li, L.; Dong, Z.Q.; Lv, F.; Guo, H.Y.; Wang, K.; Li, M.G.; Qian, Z.Y.; Ye, N.; Lin, Z.; Luo, M.C.; Guo, S.J. Pinning effect of lattice Pb suppressing lattice oxygen reactivity of Pb-RuO_2 enables stable industrial-level electrolysis. *Nat. Commun.* **2024**, *15*, 9774.
DOI: 10.1038/s41467-024-53905-y

[S3] Yang, J.; An, K.; Yu, Z.; Qiao, L.; Cao, Y.; Zhuang, Y.; Liu, C.; Li, L.; Peng, L.; Pan, H. Effect of cation and anion vacancies in ruthenium oxide on the activity and stability of acidic oxygen evolution. *ACS Catal.* **2024**, *14*, 17739-17747.
DOI: 10.1021/acscatal.4c02779

[S4] Xu, J.; Kao, C.-C.; Shen, H.F.; Liu, H.; Zheng, Y.; Qiao, S.Z. $Ru_{0.1}Mn_{0.9}O_x$ electrocatalyst for durable oxygen evolution in acid seawater. *Angew. Chem. Int. Ed.* **2024**, *n/a*, e202420615.
DOI: 10.1002/anie.202420615

[S5] Wu, H.; Chang, J.; Yu, J.; Wang, S.; Hu, Z.; Waterhouse, G.I.N.; Yong, X.; Tang, Z.; Chang, J.; Lu, S. Atomically engineered interfaces inducing bridging oxygen-mediated deprotonation for enhanced oxygen evolution in acidic conditions. *Nat. Commun.* **2024**, *15*, 10315.
DOI: 10.1038/s41467-024-54798-7

[S6] Wang, Y.; Lei, X.; Zhang, B.; Bai, B.; Das, P.; Azam, T.; Xiao, J.; Wu, Z.-S. Breaking the Ru−O−Ru symmetry of a RuO_2 catalyst for sustainable acidic water oxidation. *Angew. Chem. Int. Ed.* **2024**, *63*, e202316903.
DOI: 10.1002/anie.202316903

[S7] Sun, Y.; Xiao, M.; Liu, F.; Gan, J.; Gao, S.; Liu, J. Oxygen Vacancy-Electron Polarons Featured InSnRuO2 Oxides: Orderly and concerted In-O_v-Ru-O-Sn substructures for acidic water oxidation. *Adv. Mater.* **2024**, 2414579. DOI: 10.1002/adma.202414579

[S8] Sun, P.P.; Qiao, Z.L.; Dong, X.B.; Jiang, R.; Hu, Z.-T.; Yun, J.; Cao, D.P. Designing 3d transition metal cation-doped $MRuO_x$ as durable acidic oxygen evolution electrocatalysts for PEM water electrolyzers. *J. Am. Chem. Soc.* **2024**, *146*, 15515-15524. DOI: 10.1021/jacs.4c04096

[S9] Song, H.; Yong, X.; Waterhouse, G.I.N.; Yu, J.; Wang, H.; Cai, J.; Tang, Z.; Yang, B.; Chang, J.; Lu, S. RuO_2–CeO_2 Lattice matching strategy enables robust water oxidation electrocatalysis in acidic media via two distinct oxygen evolution mechanisms. *ACS Catal.* **2024**, *14*, 3298-3307. DOI: 10.1021/acscatal.3c06182

[S10] Shen, Y.; Zhang, X.-L.; Qu, M.-R.; Ma, J.; Zhu, S.; Min, Y.-L.; Gao, M.-R.; Yu, S.-H. Cr dopant mediates hydroxyl spillover on RuO_2 for high-efficiency proton exchange membrane electrolysis. *Nat. Commun.* **2024**, *15*, 7861. DOI: 10.1038/s41467-024-51871-z

[S11] Ji, Q.; Tang, B.; Zhang, X.; Wang, C.; Tan, H.; Zhao, J.; Liu, R.; Sun, M.; Liu, H.; Jiang, C.; Zeng, J.; Cai, X.; Yan, W. Operando identification of the oxide path mechanism with different dual-active sites for acidic water oxidation. *Nat. Commun.* **2024**, *15*, 8089. DOI: 10.1038/s41467-024-52471-7
